# New Light-Green Thermally Activated Delayed Fluorescence Polymer Based on Dimethylacridine-Triphenyltriazine Light-Emitting Unit and Tetraphenylsilane Moiety as Non-Conjugated Backbone

**DOI:** 10.3390/polym15010067

**Published:** 2022-12-24

**Authors:** René A. Hauyon, Denis Fuentealba, Nancy Pizarro, María C. Ortega-Alfaro, Víctor M. Ugalde-Saldívar, Patricio A. Sobarzo, Jean Medina, Luis García, Ignacio A. Jessop, Carmen M. González-Henríquez, Alain Tundidor-Camba, Claudio A. Terraza

**Affiliations:** 1 Research Laboratory for Organic Polymers (RLOP), Faculty of Chemistry and of Pharmacy, Pontificia Universidad Católica de Chile, Santiago 7820436, Chile; 2 Laboratorio de Estructuras Biosupramoleculares, Facultad de Química y de Farmacia, Pontificia Universidad Católica de Chile, Santiago 7820436, Chile; 3 Facultad de Ciencias Exactas, Departamento de Ciencias Químicas, Universidad Andrés Bello, Viña del Mar 2520000, Chile; 4 Instituto de Ciencias Nucleares, Universidad Nacional Autónoma de México, Circuito Exterior, Cd. Universitaria, Coyoacán, Mexico City 04510, Mexico; 5 Departamento de Química Inorgánica y Nuclear, Facultad de Química, Universidad Nacional Autónoma de México, Circuito Exterior, Cd. Universitaria, Coyoacán, Mexico City 04510, Mexico; 6 Organic and Polymeric Materials Research Laboratory, Department of Chemistry, Universidad de Tarapacá, Arica 1000007, Chile; 7 Laboratory of Nanotechnology and Advanced Materials (LNnMA), Chemistry Department, Universidad Tecnológica Metropolitana, Santiago 8940577, Chile; 8 Programa Institucional de Fomento a la Investigación, Desarrollo e Innovación (PIDi), Universidad Tecnológica Metropolitana, Ignacio Valdivieso 2409, Santiago 8940577, Chile; 9 UC Energy Research Center, Pontificia Universidad Católica de Chile, Santiago 7820436, Chile

**Keywords:** TADF, TADF-polymer, OLED devices, tetraphenylsilane, TRPL spectroscopy

## Abstract

In the search for solution-processable TADF materials as a light emitting layer for OLED devices, polymers have attracted considerable attention due to their better thermal and morphological properties in the film state with respect to small molecules. In this work, a new polymer (*p*-TPS-DMAC-TRZ) with thermally activated delayed fluorescence (TADF) light-emitting characteristics was prepared from a conjugation-break unit (TPS) and a well-known TADF core (DAMC-TRZ). This material was designed to preserve the photophysical properties of DAMC-TRZ, while improving other properties, such as thermal stability, promoted by its polymerization with a TPS core. Along with excellent solubility in common organic solvents such as toluene, chloroform and THF, the polymer (M_n_ = 9500; M_w_ = 15200) showed high thermal stability (TDT_5%_ = 481 °C), and a T_g_ value of 265 °C, parameters higher than the reference small molecule DMAC-TRZ (TDT_5%_ = 305 °C; T_g_ = 91 °C). The photoluminescence maximum of the polymer was centered at 508 nm in the solid state, showing a low redshift compared to DMAC-TRZ (500 nm), while also showing a redshift in solution with solvents of increasing polarity. Time-resolved photoluminescence of *p*-TPS-DMAC-TRZ at 298 K, showed considerable delayed emission in solid state, with two relatively long lifetimes, 0.290 s (0.14) and 2.06 s (0.50), and a short lifetime of 23.6 ns, while at 77 K, the delayed emission was considerably quenched, and two lifetimes in total were observed, 24.6 ns (0.80) and 180 ns (0.20), which was expected from the slower RISC process at lower temperatures, decreasing the efficiency of the delayed emission and demonstrating that *p*-TPS-DMAC-TRZ has a TADF emission. This is in agreement with room temperature TRPL measurements in solution, where a decrease in both lifetime and delayed contribution to total photoluminescence was observed when oxygen was present. The PLQY of the mCP blend films with 1% *p*-TPS-DMAC-DMAC-TRZ as a dopant was determined to be equal to 0.62, while in the pure film, it was equal to 0.29, which is lower than that observed for DMAC-TRZ (0.81). Cyclic voltammetry experiments showed similarities between *p*-TPS-DMAC-TRZ and DAMC-TRZ with HOMO and LUMO energies of −5.14 eV and −2.76 eV, respectively, establishing an electrochemical bandgap value of 2.38 eV. The thin film morphology of *p*-TPS-DMAC-TRZ and DMAC-TRZ was compared by AFM and FE-SEM, and the results showed that p-TPS-DMAC-TRZ has a smoother surface with fewer defects, such as aggregations. These results show that the design strategy succeeded in improving the thermal and morphological properties in the polymeric material compared to the reference small molecule, while the photophysical properties were mostly maintained, except for the PLQY determined in the pure films. Still, these results show that p-TPS-DMAC-TRZ is a good candidate for use as a light-emitting layer in OLED devices, especially when used as a host-guest mixture in suitable materials such as mCP.

## 1. Introduction

Organic light-emitting diodes (OLEDs) are a well-established technology for full-color light-emitting displays [1] and hold promise for future applications such as skin-wearable devices [2], white-light emitting sources [3,4] and biomedical purposes [5,6].

Recently, a third generation of OLEDs that emit light through thermally activated delayed fluorescence (TADF) has emerged as a superior alternative over first- and second-generation OLEDs that use conventional fluorescence and phosphorescence as light-emitting mechanisms, respectively [7]. Spin statistics predict that, under electrical excitation, the recombination of electrons and holes will produce singlet and triplet excitons in a 1:3 ratio. Of them, only the singlet excitons can emit light in first-generation OLEDs, thus limiting the internal quantum efficiency (IQE) of the device to a maximum of 25% and, considering a typical outcoupling efficiency of 0.2 [8], it limits the external quantum efficiency (EQE) of the device to a maximum of 5%. Second-generation OLEDs enhanced the maximum IQE to 100% by allowing emission from the triplet state and singlet to triplet state conversion by intersystem crossing (ISC), but required the use of expensive metal atoms such as iridium or platinum, which increases the cost of the final product. Additionally, blue emitters containing these atoms suffer significantly from low stability, shortening the lifetime operation of the devices [9]. 

TADF molecules emit light directly via the singlet state as prompt fluorescence and harvest triplet excitons through reverse intersystem crossing (RISC), converting them into singlet excitons that emit light as delayed fluorescence, making use of both types of excitons. This mechanism increases IQE to 100% but without requiring expensive metal atoms [10,11,12,13].

Since Adachi et al. [14] reported the first non-metal TADF molecule in 2011, many small molecules using this light-emitting mechanism have been reported. All samples had high photoluminescence quantum yields (PLQY) and were used as emitters for OLEDs with high EQEs as high as OLEDs using phosphorescent small molecules [7,12,15]. These reports have led to the design of different strategies to achieve TADF in organic molecules. As a common point, the highest occupied molecular orbital (HOMO) and lowest unoccupied molecular orbital (LUMO) must be localized in the donor and acceptor moieties, respectively, ensuring low singlet-triplet energy difference (ΔE_ST_) and thus achieving a high *k*_RISC_ [13,16,17,18].

Despite the numerous studies on TADF small molecules, there have been fewer reports on TADF polymeric materials. Polymers have intrinsic advantages over small molecules, positioning them as ideal materials for in-soluble processed OLEDs. Some of these advantages are higher thermal and mechanical resistance and higher morphological stability due to the formation of smoother amorphous films, which reduces the probability of short circuits in the device due to defects in layer morphology [19,20,21].

From the reports on polymeric TADF materials used as a light-emitting layers in OLED devices, some design strategies have been suggested to improve the performance of these materials. For example, incorporating a known TADF moiety based on a small molecule into a polymeric framework could yield a TADF polymer with good photophysical properties, similar to the TADF moiety used, and superior thermal, solubility, and morphology profiles [22,23]. A promising design strategy to obtain TADF polymers is using a non-conjugating unit linked in the main chain with the TADF moiety to preserve this unit’s photophysical properties while incorporating those of polymeric materials as described earlier [19,20]. A noteworthy backbone unit is the tetraphenylsilane (TPS) unit, which has been used in the synthesis of small molecules and polymeric materials with different characteristics for phosphorescent OLEDs [24,25,26,27,28]. Its use is due to its ability to impart high thermal resistance, amorphous thin-film forming behavior to the material, along with having a high triplet energy, which would limit the loss of energy by transfers to the backbone [29]. Likewise, the TPS moiety is considered a conjugation-breaking unit due to its tetrahedral “twisted” geometry centered on the silicon atom [30]. It has been narrowly used in the field of TADF materials, and few reports have been published accordingly [30,31,32].

In this context, more work is needed to study the potential use of TPS unit as backbone conjugation-breaking moiety in TADF polymers. In this work, a new TADF polymer containing the TPS unit and a well-studied TADF small molecule (DMAC-TRZ) was synthesized and characterized. This new polymer showed emission similar to that of DMAC-TRZ and had high thermal stability and good solubility in organic solvents such as toluene, chloroform and THF, making it a good candidate for use in OLED devices as an emitting layer. 

## 2. Experimental Section

### 2.1. Materials

Chemicals reagents mentioned in this work and those used for the synthesis of the monomers were obtained from commercial sources as follows: 4-bromobenzaldehyde, benzamidine hydrochloride, 9,9-dimethyl-9,10-dihydroacridine, tri-^t^butylphosphonium tetrafluoroborate, tetrakis(triphenyl phosphine) palladium(0) and phenylboronic acid were obtained from AK Scientific Inc. (Union City, CA, USA). Copper(II) acetate monohydrate, palladium(II) acetate, *N*-bromosuccinimide (NBS), potassium carbonate, and potassium bromide were obtained from Sigma Aldrich Chemical (Milwaukee, MI, USA). Tetra-*n*-hexylammonium perchlorate was obtained from Fisher Scientific (Waltham, MA, USA). Solvents and other reagents such as *n*-hexane, toluene, dichloromethane, chloroform, tetrahydrofuran, methanol, *N*,*N*-dimethylformamide, bromobenzene and anhydrous magnesium sulfate were obtained from Merck (Burlington, MA, USA). The synthesis of 2,7-dibromo-10-(4-(4,6-diphenyl-1,3,5-triazin-2-yl)phenyl)-9,9-dimethyl-9,10-dihydroacridine (**1**) was taken from the literature [22,33] as well as for diphenylbis(4-(4,4,5,5-tetramethyl-1,3,2-dioxaborolan-2-yl)-phenyl)silane (**2**) [28]. 

### 2.2. Equipment

FT-IR spectra (KBr pellets) were recorded on a Perkin-Elmer (Fremont, CA, USA) 1310 spectrophotometer over the range of 4000–450 cm^−1^. NMR spectra were acquired on a 400 MHz spectrometer (BRUKER AVANCE III HD-400, Bremen, Germany) using CDCl_3_ as solvent and TMS as an internal standard. Asterisked peaks in the ^13^C NMR description correspond to un-observed signals in DEPT-135. Elemental analyses were performed on a Fisons EA 1108-CHNS-O instrument (Thermo Scientific, Waltham, MA, USA). The average molecular weights (M_n_ and M_w_) of *p*-TPS-DMAC-TRZ, the degree of polymerization, and polydispersity index were determined on a GPC system 150cv (Waters, Milford, CT, USA) equipped with a refractive index detector. Commercial polystyrene samples were used as standards, and THF was employed as a solvent. For this, a solution of *p*-TPS-DMAC-TRZ (c = 0.5 mg/mL) was prepared, and then 100 μL were injected at 1 mL/min in a MesoPore Column 300 × 75 mm. Thermogravimetric analyses (TGA) were carried out in a Mettler-Toledo (Greifensee, Switzerland) TA-3000 calorimetric system equipped with a TC-10A processor and a TG-50 thermobalance with a Mettler MT5 microbalance. Six milligrams of sample were placed in an alumina sample holder and analyzed between 25 °C and 850 °C with a heating rate of 20 °C/min under nitrogen flow. Glass transition temperature was obtained with a Mettler-Toledo (Greifensee, Switzerland) DSC 821 calorimetric system (20 °C/min under nitrogen flow) after the second heating scan. UV-vis and photoluminescent spectra were acquired on an Edinburgh Instruments FS5 fluorimeter (Edinburgh, Scotland) equipped with a Xenon lamp at 25 °C temperature in solutions of toluene, 1,4-dioxane, THF, chloroform and dichloromethane. The concentration of these solutions was adjusted to obtain an absorbance of 0.1 before proceeding to photoluminescent measurements. In this same fluorimeter, the CIE (Commission Internationale de l’éclairage) coordinates of each emission in the different solvents used were obtained. For all measurements carried out in the absence of oxygen, a 15 min N_2_ purge was applied to each solution, and a quartz cell (path length = 1 cm) equipped with a Luer-Lock cap and a rubber membrane was used. PLQY determination in solution were carried out at room temperature using quinine bisulfate in 0.5 M H_2_SO_4_ (PLQY = 0.546) as standard [34]. The absorption of each solution was adjusted to A = 0.1, and the same cuvettes were used. In addition, the spectrometer parameters (emission wavelength, excitation wavelength, slits and speed) were maintained constant throughout the measurements. The fluorescence spectra were integrated (I), and the relative fluorescence quantum yields were calculated according to the formula:

$$\mathrm{PLQY}=\frac{I_{\mathrm{FL}}}{I_{\mathrm{FL}}^{s}}\times \frac{n^{2}}{n_{s}^{2}}{\times \mathrm{PLQY}}^{s}$$

where $I_{\mathrm{FL}}$,$\;I_{\mathrm{FL}}^{s}$ = fluorescence intensities (index s refers to the quinine bisulfate 0.5 M H_2_SO_4_ standard), and n,${\;n}_{s}$ = refractive indices.

Photoluminescence lifetime measurements in solution were carried out using the time-correlated single photon counting (TCSPC) method in a LifeSpecII fluorescence lifetime spectrometer (Edinburgh Instruments, Edinburgh, Scotland) equipped with an Edinburgh Instruments picosecond pulsed diode laser, model EPL-375 (wavelength = 375.6 nm, pulse width = 61.1 ps, average power = 5 mW). For all lifetime measurements, the obtained decays were fitted by tail fit due to the long lifetimes obtained and without the use of an IRF. The equation from which the lifetimes were obtained is as follows:

$$I\left(t\right)=\overset{n}{\underset{i=1}{\sum }}A_{i}e^{-\frac{t}{\tau_{i}}}$$

where I (t) = photoluminescence intensity over time, n = number of lifetimes, t = time, A_i_ = pre-exponential coefficient, and τ = lifetime.

The ratio or contribution of each lifetime to total photoluminescence intensity was obtained directly from the fluorimeter using the above equation, but can also be manually calculated using the following formula: [33]

$$\mathrm{photoluminescence}\;\mathrm{intensity}\;\mathrm{ratio}=\frac{A_{i}{\;\times \;\tau }_{i}}{\sum_{i=1}^{n}A_{n}{\;\times \;\tau }_{n}}$$

where n = number of lifetimes, A_i_ = pre-exponential coefficient, and τ = lifetime.

Additionally, solid-state PL emission of *p*-TPS-DMAC-TRZ was obtained with the same equipment by depositing the polymer by the drop-casting technique from a dichloromethane solution. 

Photoluminescence lifetime measurements in solid-state were obtained by the TCSPC method using a PicoQuant FluoTime 300 (Berlin, Germany) fluorescence lifetime spectrometer at room temperature or in EtOH/MeOH glass (4:1 *v*/*v*) at 77 K, equipped with a sub-nanosecond pulsed laser 405 nm as pulsed light source (FWHM ~500 ps; average power 2 mW). Absolute fluorescence quantum yield in solid-state of *p*-TPS-DMAC-TRZ was measured in a FluoroMax 4CP (Horiba Jovin Yvon, Longjumeau, France) spectrofluorometer equipped with the Quanta-Phi integrating sphere. Electrochemical properties of *p*-TPS-DMAC-TRZ were investigated by cyclic voltammetry performed in tetrahexylammonium perchlorate 0.2 M dichloromethane solution on a CH Instruments bipotentiostat, model CHI760E (Austin, TX, USA), using a three-electrode cell with glassy carbon as working electrode, platinum wire as a counter-electrode and Ag/AgCl as the reference electrode at a scan rate of 100 mV/s. The half-wave potential of the ferrocenium/ferrocene ion couple (Fc/Fc^+^) under these conditions using a AgCl/Ag reference electrode was 0.62 V, and this system was used as internal reference. The electrochemical determinations were carried out with compensation of ohmic drop. The topography of the films of DMAC-TRZ and *p*-TPS-DMAC-TRZ were obtained with a CoreAFM from Nanosurf Inc. (Woburn, MA, USA) in contact mode, using a tip of PPP-XYCONTR (Nanosensors, Neuchatel, Switzerland). Further, a field emission scanning electron microscope (FE-SEM) model GeminiSEM 360 (Carl Zeiss AG, Oberkochen, Germany) was utilized to visualize the material surface.

### 2.3. Thin Film Preparation for AFM and FE-SEM Analysis

The DMAC-TRZ and *p*-TPS-DMAC-TRZ films were prepared following a standard procedure: round glass substrates (~1 cm in diameter) were washed 3 times using water/detergent solution and for 5 min using an ultrasonic bath at room temperature. After that, the substrates were cleaned with isopropyl alcohol to remove detergent traces and dried in a vacuum oven at 40 °C for 30 min. Then, 20 µL of a 5:1 (% *v*/*v*) isopropyl alcohol and PEDOT:PSS solution, previously filtered using a 0.45 µm syringe filter, were spin-coated at 1500 rpm for 9 s and 5000 rpm for 30 s, and dried in a vacuum oven at 34 °C for 30 min. After this, 20 µL of a DMAC-TRZ or *p*-TPS-DMAC-TRZ chloroform solution, with a concentration of 5 mg/mL, were spin-coated at the same speed used for the PEDOT:PSS layer and dried in a vacuum oven at 34 °C for 15 min. 

### 2.4. Synthesis of p-TPS-DMAC-TRZ

In a 50 mL two-neck round-bottom flask equipped with a condenser, 435.2 mg (0.645 mmol) of the dibromide monomer (**1**), 379.7 mg (0,645 mmol) of the diboronic ester monomer (**2**), 15 mg (0.046 mmol) of tetrabutylammonium bromide and 4.2 mL of toluene were added. The mixture was purged under a steady stream of N_2_ for 30 min and then, 45.0 mg (0.039 mmol) of tetrakis(triphenylphosphine) palladium (0) and 4.2 mL of a 2 M aq. solution of potassium carbonate were added. The mixture was stirred and refluxed for 36 h, after which 39.34 mg (0.323 mmol) of phenylboronic acid was added, and the system was refluxed for another 12 h. Then, 50.66 mg (0.323 mmol) of bromobenzene was added to the mixture and refluxed for 12 h. The system was then cooled to room temperature, poured into 300 mL of methanol, and stirred for 2 h. The obtained solid was filtered and washed sequentially with a Soxhlet apparatus using methanol and acetone. The crude product was recovered from chloroform solution and then concentrated in a rotary evaporator. Subsequently, 5 mL of chloroform were added to dissolve the product and precipitate it from methanol. The obtained solid was recovered via centrifugation and then dried in a vacuum oven at 100 °C to constant mass to afford 368 mg of a green solid as the pure product (67% yield). 

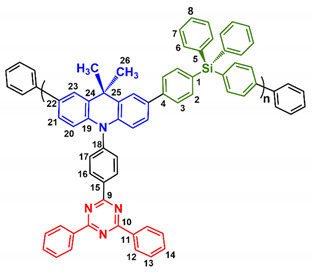



FT-IR (KBr, ν, cm^−1^): 3062, 3044, 3028 (C-H arom.); 2963 (C-H aliph.); 1590, 1520 (C=C arom.); 1471 (C=N arom.); 1365 (C-N); 1111 (Si-C), 845 (arom. 1,2,4-tri-subst.) 806 (arom. *p*-subst.); 771 (arom. *mono*-subst.). ^1^H NMR (CDCl_3_, δ, ppm): 1.83 (s, 6H, H26); 6.47 (d, *J* = 8.1 Hz, 2H, H20); 7.27 (d, *J* = 8.0 Hz, 2H, H21); 7.34–7.46 (m, 6H, H7, H8); 7.50–7.70 (m, 20H, H2, H3, H6, H13, H14, H17); 7.77 (s, 2H, H23); 8.70–8.86 (m, 4H, H12); 8.95–9.10 (m, 2H, H16). ^13^C NMR (CDCl_3_, δ, ppm): 31.92 (C26); 36.55* (C25); 114.83 (C20); 124.62 (C23); 125.45 (C21); 126.15 (C3); 128.04 (C7); 128.87 (C13); 129.18 (C12); 129.73 (C8); 130.60* (C24); 131.61 (C17); 131.87 (C16); 132.24* (C1); 132.86 (C14); 133.71* (C22); 134.57* (C5); 136.20* (C4); 136.57, 137.04 (C2, C6); 140.15* (C19); 142.31* (C11), 142.37* (C15); 145.13* (C18); 171.17* (C9); 172.03* (C10). ^29^Si NMR (CDCl_3_, δ, ppm): −14.46. GPC (THF, PS, g/mol) M_n_ = 9500; M_w_ = 15200; PDI = 1.6. 

## 3. Results and Discussion

### 3.1. Synthesis and Characterization of Monomers

**Scheme 1:**
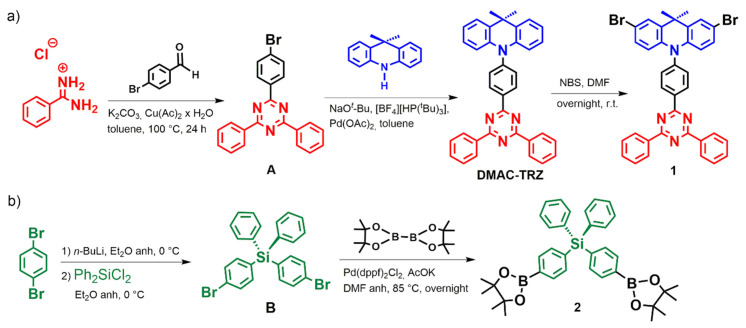
Synthetic procedures to obtaining the monomers **1** (route a) and **2** (route b).

The preparation of both monomers was developed following already reported routes (Scheme 1). The colors in the structure of the precursors, monomers and polymer reflect the roles those units play in *p*-TPS-DMAC-TRZ. The red color is used for the acceptor unit, the blue color is used for the donor moiety, and the green color is used for the conjugation-breaking backbone unit. To obtain 2,7-dibromo-10-(4-(4,6-diphenyl-1,3,5-triazin-2-yl)phenyl)-9,9-dimethyl-9,10-dihydro acridine (**1**), a three-step route was followed. The first one was an oxidative copper-catalyzed cyclization of amino(phenyl)methaniminium chloride and 4-bromobenzaldehyde (Scheme 1a) [35]. This reaction allowed an efficient synthesis (64% yield) of 2-(4-bromophenyl)-4,6-diphenyl-1,3,5-triazine (**A**) compared to other previously reported synthetic routes that require more steps, additional time and with overall lower yields [36,37]. The second step consisted of a Buchwald–Hartwig cross coupling between the acceptor fragment **A** and the donor fragment, 9,9-dimethyl-9,10-dihydroacridine [33] to obtain 2-(4-bromophenyl)-4,6-diphenyl-1,3,5-triazine (**DMAC-TRZ**) in 85% yield with minor modifications. Namely, the use of sodium ^t^butoxide instead of potassium carbonate as the base and the use of the tetrafluoroborate salt of the tri-^t^butylphosphine ligand. These changes, but most probably, the change in the base used made it possible to decrease the reaction time from 2 days to 2.5 h. This fact is in accordance with the proposed mechanism [38,39] for the reaction, in which the coordination of the amine-nitrogen atom to the palladium catalyst is followed by deprotonation of this center to afford a deprotonated amine-coordinated palladium complex that undergoes a reductive elimination affording the product. This step could be accelerated by the use of a stronger base such as sodium ^t^butoxide and thus accelerate the whole reaction. The final synthetic step of the route consisted of a simple aromatic dibromination using NBS in DMF as solvent at room temperature (67% yield) [22].

The synthesis of diphenylbis(4-(4,4,5,5-tetramethyl-1,3,2-dioxaborolan-2-yl)-phenyl)silane (**2**) required two separate reactions (Scheme 1b), in which the first one corresponded to a nucleophilic substitution over dichlorodiphenylsilane by two equivalents of the in situ formed (4-bromophenyl)lithium intermediate [40]. The formed dibromide derivative **B** (bis(4-bromophenyl)diphenylsilane, 70% yield) was subsequently modified at 85 °C under Miyaura-borylation conditions to obtain the corresponding monomer in 75% yield [28].

The structure of both monomers was confirmed by spectroscopic techniques (IR and NMR). The FT-IR spectrum of (**1**) showed the bands associated with the C-N stretches of the s-triazine (1523 cm^−1^ and 1366 cm^−1^) and acridine (1390 cm^−1^) moieties. For (**2**), the FT-IR spectrum revealed the Si-C (1150 cm^−1^ and 712 cm^−1^), C-B (1070 cm^−1^) and B-O (1360 cm^−1^) stretches. 

**Figure 1:**
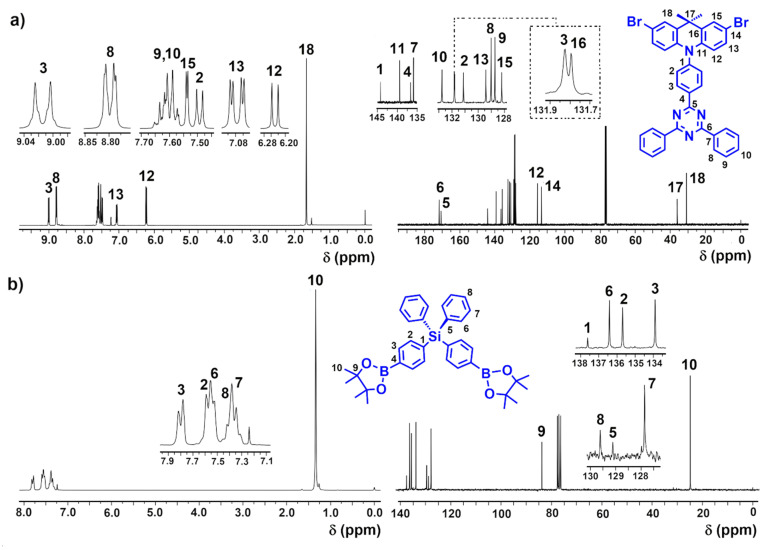
^1^H (left) and ^13^C (right) NMR spectra (CDCl_3_) of monomers. (**a**) 2,7-dibromo-10-(4-(4,6-diphenyl-1,3,5-triazin-2-yl)phenyl)-9,9-dimethyl-9,10-dihydroacridine (**1**) and (**b**) diphenylbis(4-(4,4,5,5-tetramethyl-1,3,2-dioxaborolan-2-yl)-phenyl)silane (**2**).

Both ^1^H NMR spectra (Figure 1, left side) showed complex aromatic systems, which, despite the overlapping of some signals, were resolved with the help of 2D NMR techniques. The deshielding action of the triazine ring in (**1**) was evident when observing the displacements of H3 and H8 (δ > 8.5 ppm), and also observed in the ^13^C NMR spectrum for the C5 and C6 nuclei (δ > 170 ppm) (Figure 1a, right). It is normal, when examining a ^13^C NMR spectrum of (**2**), to observe only six of the eight expected aromatic signals [28,41], excluding nuclei C5 and C8. However, on this occasion, the presence of C5 was also detected, presenting a chemical shift close to 129 ppm (Figure 1b, right).

### 3.2. Synthesis and Structural Characterization of p-TPS-DMAC-TRZ

**Scheme 2:**
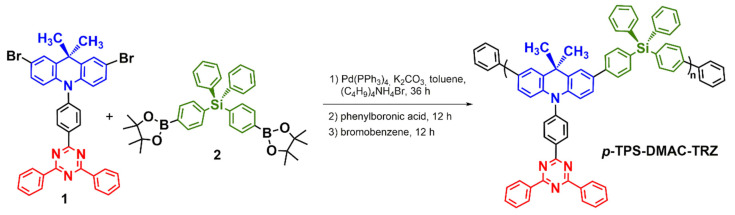
Synthetic route for *p*-TPS-DMAC-TRZ.

The synthesis of the polymer containing triphenyltriazine (TRZ), dimethylacridine (DMAC) and TPS moieties (*p*-TPS-DMAC-TRZ) was carried out by a poly-Suzuki-Miyaura cross-coupling (Scheme 2) between the dibromide (**1**) and the diboronic ester monomer (**2**) according to a previously reported procedure with minor modifications [42,43,44]. The polymer was end-capped and purified by Soxhlet extractions to provide chemical stability and remove catalyst traces, by-products, or oligomeric species, respectively.

**Figure 2:**
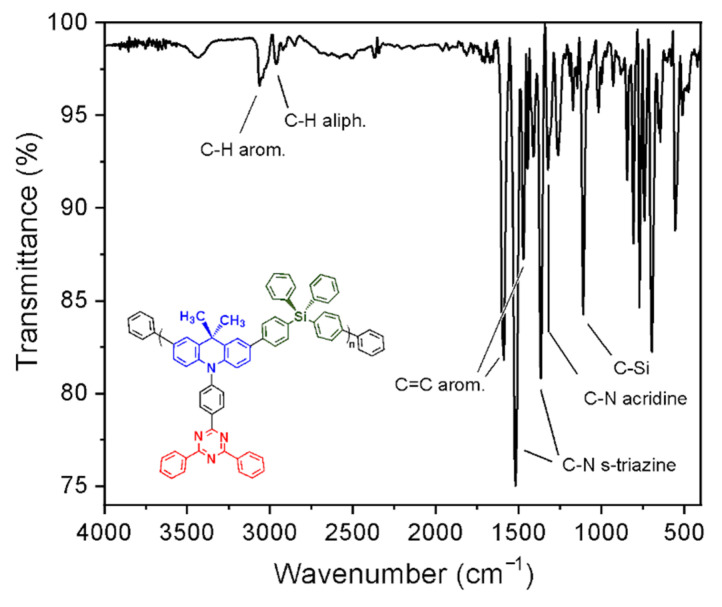
FT-IR spectrum for *p*-TPS-DMAC-TRZ.

The spectroscopic information obtained from FT-IR and NMR analyses corroborated the structure and high purity of the polymer. The FT-IR spectrum (Figure 2) showed absorption bands related to the aromatic backbone of the polymer, such as the C-H bond stretching just above 3000 cm^−1^ and the C=C bond stretching at 1589 and 1483 cm^−1^. Specific absorption bands were observed for the triphenyltriazine unit, related to C-N bond stretching at 1520 cm^−1^ and 1366 cm^−1^, along with the absorption band corresponding to the Si-C bond stretching, which was observed at 1111 cm^−1^ in accordance with spectra for previously reported TPS-containing polymers [40]. 

**Figure 3:**
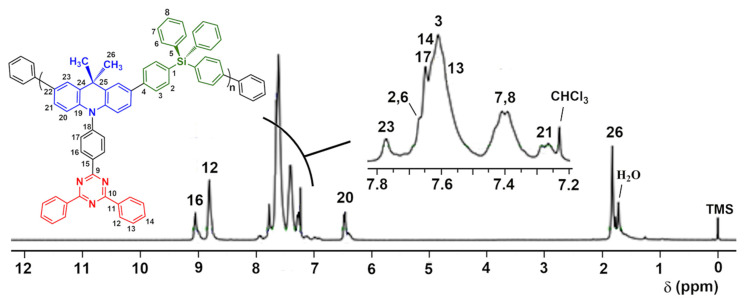
^1^H NMR spectrum (CDCl_3_) of *p*-TPS-DMAC-TRZ.

In the ^1^H NMR spectrum (Figure 3), the methyl groups of the dimethylacridine moiety were observed at 1.83 ppm as a singlet with an integral equal to 6H. In addition, 44 aromatic H nuclei were observed between 6.47 ppm and 9.10 ppm, being H12 and H16 deshielded due to the electron-withdrawing effect of the triazine ring. In contrast, the H20 was shielded due to the electron-donating effect of the N lone pair in the dimethylacridine unit. 

**Figure 4:**
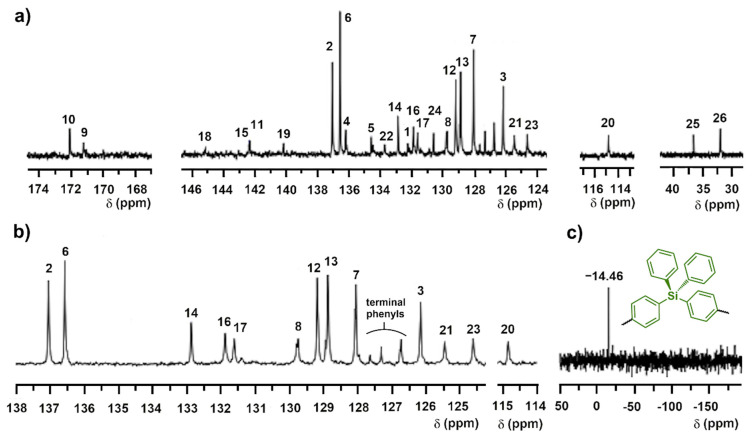
^13^C NMR spectrum (**a**), aromatic zone dept-135° (**b**) and ^29^Si NMR spectrum (**c**) for *p*-TPS-DMAC-TRZ in CDCl_3_.

The ^13^C-NMR spectrum (Figure 4a) showed the methyl groups and the quaternary carbon atom of the dimethylacridine fragment at 31.92 ppm and 36.55 ppm, respectively. Two signals corresponding to the *s*-triazine C-nucleus were observed at 171.19 (C9) and 172.03 (C10) ppm. The 22 other signals were related to the aromatic C-nuclei of the polymeric repeating unit (Figure 4b), while signals between 126 ppm and 128 ppm could be attributed to the phenyl end-groups. The ^29^Si-NMR spectrum (Figure 4c) showed the characteristic Si signal of the TPS unit at −14.46 ppm [40,45,46].

**Figure 5:**
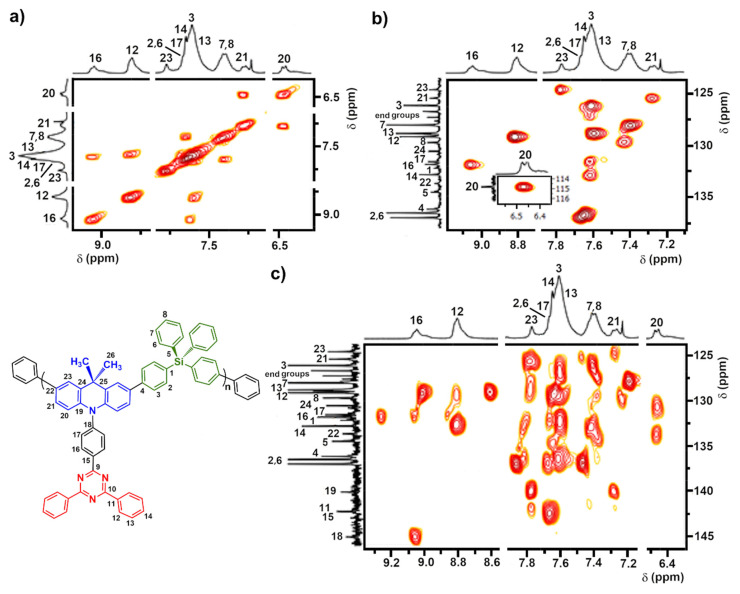
Aromatic zone 2D-NMR spectra of *p*-TPS-DMAC-TRZ. COSY (**a**), HSQC (**b**), and HMBC (**c**).

Employing 2D-NMR spectrum (COSY, HSQC, and HMBC), the ^1^H and ^13^C nuclei were fully assigned for *p*-TPS-DMAC-TRZ. COSY spectrum (Figure 5a) showed a correlation between vicinal H-nuclei such as H20-H21 that belong to the dimethylacridine moiety. HSQC (Figure 5b) spectrum showed a correlation between bonded H and C nuclei, such as H20-C20 being the most shielded aromatic nuclei. This spectrum also showed that the three signals observed between C3 and C7 (Figure 3b) had a weak correlation with the multiplet at 7.50–7.70 ppm in the ^1^H NMR spectrum, which was attributed to the phenyl end-groups. HMBC spectrum (Figure 5c) was a valuable tool for assigning quaternary C nuclei by observing the correlation between H and C nuclei at three bond distance, and sometimes two bond distance, such as H16-C18 that belong to the triphenyltriazine unit. 

### 3.3. Solubility, Molecular Weights and Thermal Properties

The solubility of the polymer was tested qualitatively using 5 mg of product and 0.5 mL of the respective solvent. At room temperature, the polymer was soluble in common organic solvents such as chloroform, dichloromethane, chlorobenzene, THF, and toluene, which is highly desirable for thin-film formation from solutions. 

Apparent molecular weight determination of *p*-TPS-DMAC-TRZ was carried out by GPC technique using THF as a solvent based on a calibration curve using polystyrene standards. *p*-TPS-DMAC-TRZ showed a moderate molecular size (M_n_ = 9500 g/mol and M_w_= 15,200 g/mol), corresponding to an average degree of polymerization (DP_n_) of 11. A relatively low polydispersity index (PDI = 1.6) was obtained, ensuring a material with a homogeneous composition and a well-defined structure that allowed its accurate structural characterization [18]. This fact, in combination with the polymerization yield, can be attributed to the success of the purification process by Soxhlet treatment and subsequent reprecipitation of the polymeric material.

**Figure 6:**
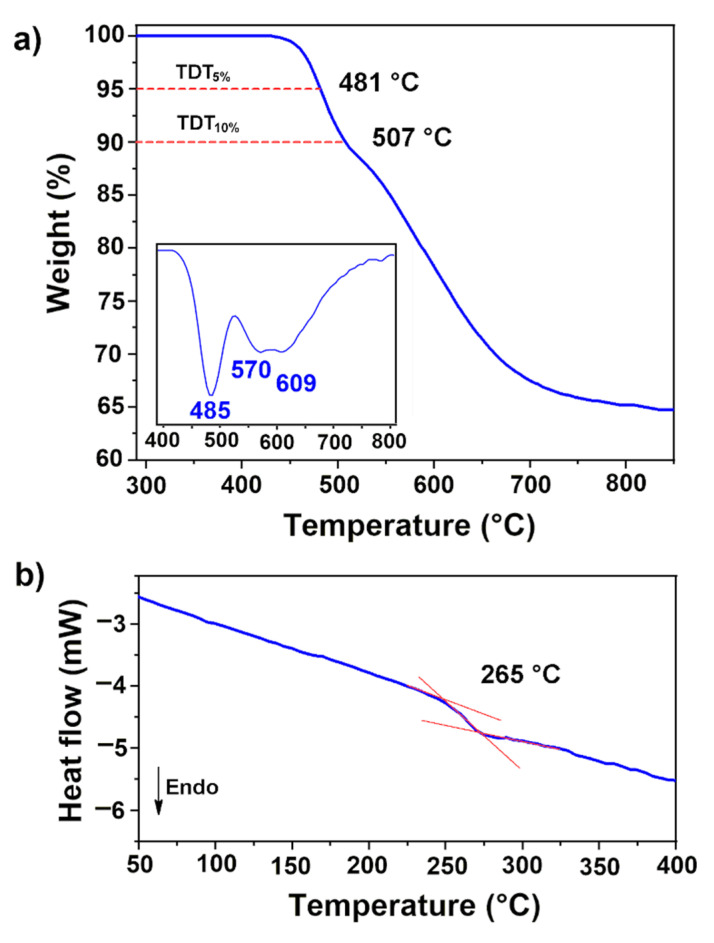
TGA and DTGA curves (**a**) and DSC traces (**b**) of *p*-TPS-DMAC-TRZ.

Thermal properties of *p*-TPS-DMAC-TRZ were evaluated using TGA and DSC techniques. The TGA curve (Figure 6a) showed a complex decomposition process where the temperature at which 5% and 10% of *p*-TPS-DMAC-TRZ mass is lost (TDT_5%_ and TDT_10%_, respectively) was 481 °C (TDT_5%_) and 507 °C (TDT_10%_), making it a highly thermally stable material. The elevated thermal resistance of this polymer can be attributed to its high aromatic content, considering that DMAC-TRZ evidenced a TDT_5%_ of 305 °C [33]. The improved thermal stability of the polymer over the TADF small molecule was an expected result due to the greater molecular size of the polymeric material. Additionally, the TPS unit contributed to this parameter with its high aromatic content [29]. 

The DSC traces of *p*-TPS-DMAC-TRZ (Figure 6b) showed a glass transition temperature (T_g_) value of 265 °C, which is higher than that of DMAC-TRZ (91 °C [33]), and no melting transitions, indicating its amorphous nature. This high T_g_ value is an essential feature for materials to be used in OLEDs since it correlates with stable film morphology and thus, a potentially longer operational lifetime of the devices [24,47,48].

### 3.4. Photophysical Properties 

**Figure 7:**
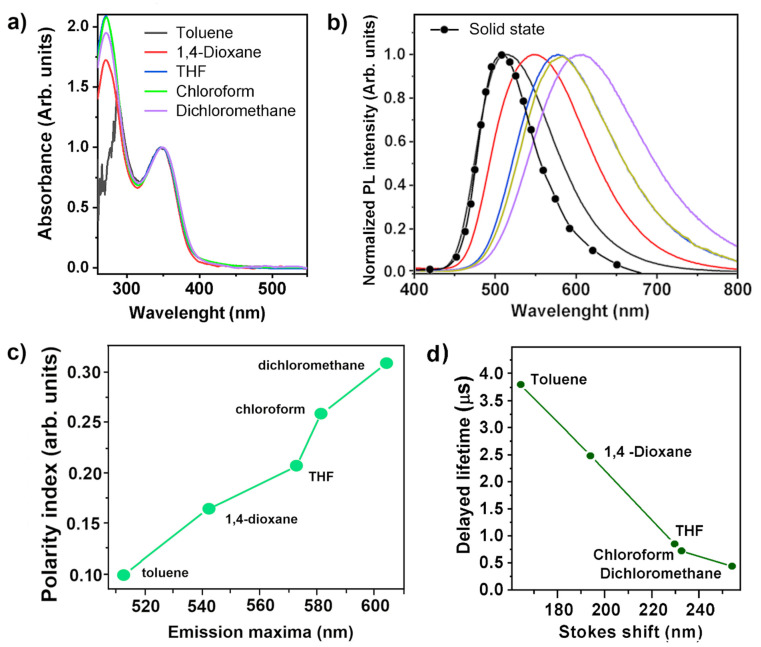
Absorption (**a**) and photoluminescence (**b**) spectra of *p*-TPS-DMAC-TRZ. (**c**) Polarity index of the solvent versus the emission maxima of *p*-TPS-DMAC-TRZ in solution of the respective solvent. (**d**) Delayed lifetime as function of Stokes shift for *p*-TPS-DMAC-TRZ solution in different solvents.

The photophysical properties of *p*-TPS-DMAC-TRZ were evaluated by UV-vis absorption, fluorescence, and time-resolved photoluminescence (TRPL) spectroscopies. UV-vis spectra in solution (Figure 7a) showed an absorption band below 300 nm in all solvents except toluene, corresponding to π-π* transitions from the aromatic units of the polymer. Around 350 nm, an absorption band corresponding to the transition due to the interaction between the DMAC donor unit and the TRZ acceptor unit in the TADF core was observed [33]. In DMAC-TRZ, this absorption band was weak and difficult to observe; however, in *p*-TPS-DMAC-TRZ the band was clearly visible, and it was observed that the absorption maxima did not shift, as the polarity of the solvent in which it was measured varied. This means that the ground state structure of the *p*-TPS-DMAC-TRZ and, consequently, of the TADF DMAC-TRZ core, has a relatively low polarity in which the weak conjugation of the donor and acceptor units does not create a strong dipolar structure. This is in contrast to known solvatochromic dyes in which the highly polar structure, where donor and acceptor units are strongly conjugated, leads to UV-vis absorption solvatochromism [49]. This is an important feature of TADF materials, as the low conjugation of donor and acceptor units leads to efficient localization of the HOMO and LUMO orbitals in the donor and acceptor fragments, respectively. This increases the rate constant of the RISC process (*k*_RISC_) and thus the efficiency of the TADF light emission mechanism.

**Figure 8:**
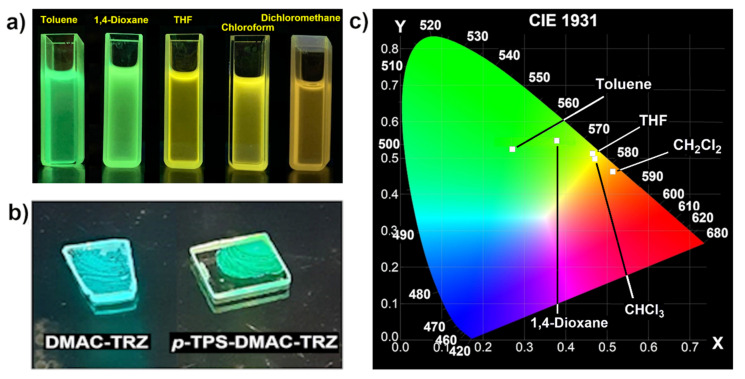
(**a**) UV-light irradiation (312 nm) behavior of *p*-TPS-DMAC-TRZ in several solvents. (**b**) Solid-state photoluminescence of DMAC-TRZ and *p*-TPS-DMAC-TRZ under the same wavelength. (**c**) Chromaticity coordinates of *p*-TPS-DMAC-TRZ emission in solution with different solvents.

Photoluminescence spectra (Figure 7b) were recorded by exciting the polymer at the wavelength maximum of the absorption band of the DMAC-TRZ interaction, showing a remarkable bathochromic shift of the emission maxima with increasing solvent polarity (Table 1). This effect is easily observed when solutions of *p*-TPS-DMAC-TRZ in the solvents studied are irradiated under UV light (312 nm), and different colors are observed, depending on the emission maxima measured in each solvent (Figure 8a). The observed correlation between the solvent polarity index and the emission maxima of the p-TPS-DMAC-TRZ in solution of the respective solvent [49] (Figure 7c) evidenced an increased stabilization of the excited state with increasing solvent polarity, which is indicative of a highly polar excited state, as opposed to the ground state structure. This evidenced the intramolecular charge transfer (ICT) characteristic of the excited state that is responsible for the light emission from *p*-TPS-DMAC-TRZ. This fact is consistent with the TADF mechanism in which the first singlet state (S_1_) from which the light emission is derived has an ICT behavior due to the intense interaction between the donor and acceptor fragments in the excited state [12,33,50]. 

To compare the emitted light color of both materials, drop-casted films of DMAC-TRZ and *p*-TPS-DMAC-TRZ onto quartz substrates were irradiated under 312 nm UV-light (Figure 8b). The color observed in both cases correlated to the measured emission maxima recorded by the fluorimeter. When comparing the emission maxima of *p*-TPS-DMAC-TRZ in the solid state and DMAC-TRZ (neat film), a difference of 8 nm was observed. This bathochromic shift could be related to the extended conjugation of the TADF moiety in the polymer due the phenyl group provided by the TPS unit, which is directly bonded to the TADF core [42]. Figure 8c shows a 2D chromaticity diagram plotting the CIE coordinates of *p*-TPS-DMAC-TRZ emission in toluene. These coordinates were X = 0.27 and Y = 0.52, clearly indicating green light emission.

**Figure 9:**
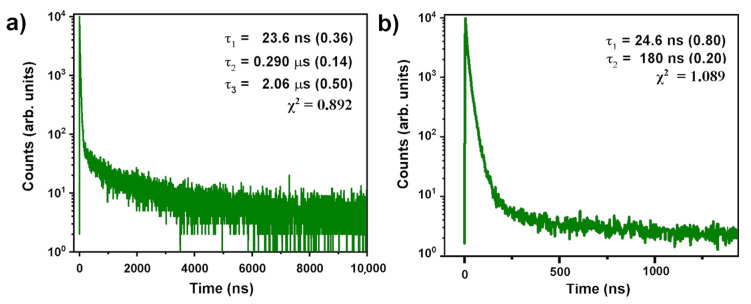
Time-resolved photoluminescence curve of *p*-TPS-DMAC-TRZ at 298 K (**a**) and 77 K (**b**). Pre-exponential coefficients: (**a**) A_1_ = 1462; A_2_ = 47.2; A_3_ = 23.0; (**b**) A_1_ = 5934; A_2_ = 200.6.

The TRPL decay of a *p*-TPS-DMAC-TRZ thin-film was obtained at 77 K and 298 K. At 298 K (Figure 9a), two clear components of the emission decay were observed: a fast component with a lifetime (τ_298 K_) of 23.6 ns and a delayed emission, which showed two lifetimes, 290 ns and 2.06 μs. The observation of two relatively long lifetimes instead of only one could be attributed to a partial quenching process of the long-lived triplet state due to concentration-caused quenching.

Due to limited energy for the occurrence of the RISC process, the TADF behavior of the polymer was clearly observed in the considerable decrease in the delayed component of the emission when measured at 77 K (Figure 9b). Thus, only 20% of light emission came from delayed fluorescence with a lifetime (τ_77 K_) of 180 ns. In contrast, the other 80% came from prompt fluorescence with a lifetime of 21.5 ns. 

It has been reported that DMAC-TRZ small molecule neat films have a PLQY of 0.83 [33]. *p*-TPS-DMAC-TRZ exhibited a significant decrease in this parameter (0.29, Table 1) but in the range of similar TADF polymers (0.28–0.37) [51]. However, a 1% mixture of p-TPS-DMAC-TRZ in 1,3-bis(*N*-carbazolyl)benzene (mCP) as a host:guest mixture showed a PLQY of 0.62, which was more than a twofold increase compared to the PLQY of the neat film. These results show that considerable exciton quenching events occur in pure polymer films, which could initially be related to aggregation-induced quenching.

**Figure 10:**
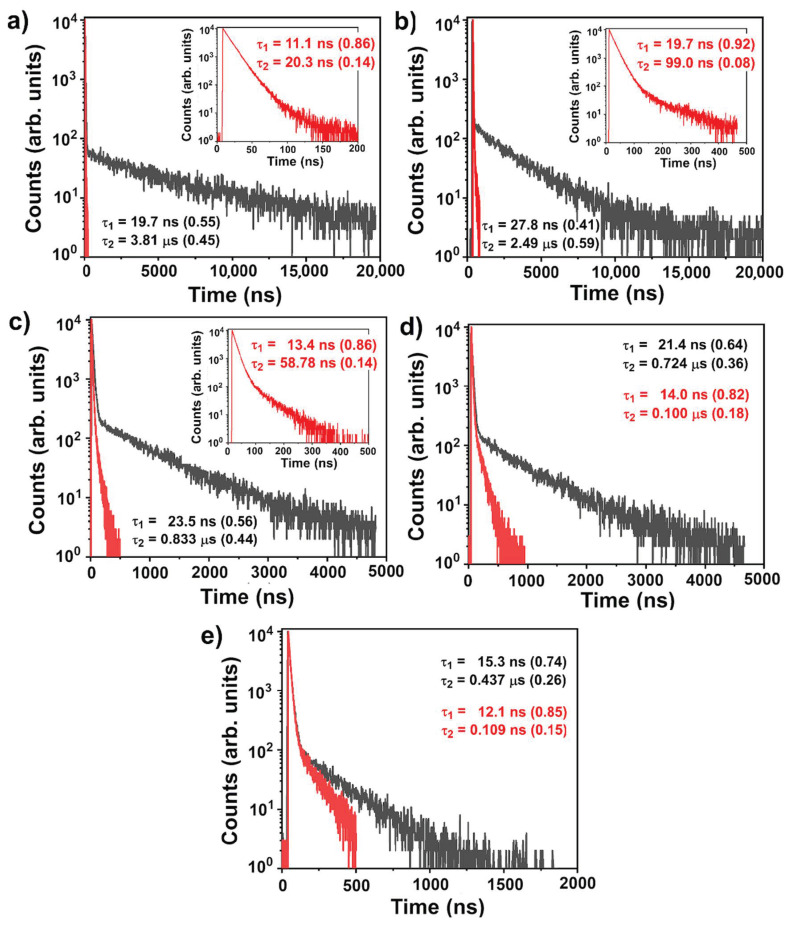
TRPL results carried out in N_2_-purged (black line) and in air (red line) for solutions of *p*-TPS-DMAC-TRZ in toluene (**a**), 1,4-dioxane (**b**), THF (**c**), chloroform (**d**), and dichloromethane (**e**). Chi-squared value (χ^2^): (**a**) N_2_ = 1.131; Air = 1.113; (**b**) N_2_ = 1.104; Air = 1.169; (**c**) N_2_ = 1.141; Air = 0.928; (**d**) N_2_ = 1.017; Air = 0.936; and (**e**) N_2_ = 0.879; Air = 1.188. Pre-exponential factors: (**a**) N_2_: A_1_ = 9649, A_2_ = 40.9; Air: A_1_ = 8976, A_2_ = 809.4. (**b**) N_2_: A_1_ = 9756, A_2_ = 155.8; Air: A_1_ = 9679, A_2_ = 157.5. (**c**) N_2_: A_1_ = 9405, A_2_ = 206.6; Air: A_1_ = 9611, A_2_ = 337.2 (**d**) N_2_: A_1_ = 9455, A_2_ = 155.9; Air: A_1_ = 9824, A_2_ = 305.2 (**e**) N_2_: A_1_ = 9475, A_2_ = 117.8; Air: A_1_ = 9863, A_2_ = 197.3.

To better understand the dynamics of the TADF process occurring in *p*-TPS-DMAC-TRZ and to test whether aggregation-induced quenching was occurring, TRPL measurements were performed in N_2_-purged and in-air solutions of the five solvents (toluene, 1,4-dioxane, THF, chloroform, and dichloromethane) in which steady-state PL emission was recorded (Figure 10). 

Oxygen is a species known to quench the excited state of triplets and, since the TADF process involves the participation of triplets, it is to be expected that a difference in emission parameters, such as lifetime and PLQY, would be observed. In fact, in all five solvents studied, the delayed component of the emission was clearly quenched, as could be seen in the decays and emission lifetimes, where no long lifetime was observed in toluene, while in 1,4-dioxane and THF, and in chloroform and dichloromethane, only a slightly long lifetime was recorded in the 0.1 μs range, with a low contribution to the overall photoluminescence intensity. However, in N_2_-purged solutions, a clear delayed component was observed with long lifetimes up to 3.81 μs in toluene, and in all solvents the delayed emission ratio was higher than in-air solutions, demonstrating the involvement of triplet excited states in the photoluminescence process, as expected for a TADF material.

PLQY determinations of *p*-TPS-DMAC-TRZ in solution were obtained using a 0.5 M quinine sulfate standard in sulfuric acid (PLQY = 0.546) and comparing the PL curve integral, both in-air and N_2_-purged versus the PL curve integral of the emission of the standard (Table 1). The results show that the PLQY of *p*-TPS-DMAC-TRZ changed significantly when the solution was purged with N_2_ compared to the solution in-air; however, this change was not uniform across all solvents, where its polarity could have influenced the emission, especially in the delayed component. In the toluene purged with N_2_ solution, the PLQY of *p*-TPS-DMAC-TRZ was 0.45; however, in-air, this parameter decreased to 0.25, indicating a partial quenching of the emission. When the prompt and delayed emission ratios were taken from the N_2_-purged TRPL decay, 0.55 and 0.45, respectively, the delayed PLQY and prompt PLQY could be obtained using the total PLQY, resulting in a delayed PLQY of 0.20 and a prompt PLQY of 0.25, which in the latter case, is the same PLQY recorded for the toluene-in-air solution of *p*-TPS-DMAC-TRZ. These results correlated well in four of the five solvents used, indicating that in-air, mainly prompt fluorescence was observed and was responsible for the light emission from the polymer, while in the absence of oxygen, both fast and delayed emissions were observed, increasing the total PLQY.

The contribution of prompt and delayed fluorescence components was not uniform in all solvents, and there could be a polarity effect involved. When comparing the *p*-TPS-DMAC-TRZ emission time obtained from toluene with those from 1,4-dioxane, THF, chloroform and dichloromethane, there was a significant decrease in the delayed emission lifetime when the solvent polarity increased (3.81 μs, 2.49 μs, 0.833 μs, 0.724 μs, and 0.437 μs, respectively) as can be observed in Figure 7d, where the increasing Stokes shift for *p*-TPS-DMAC-TRZ in each solvent could be used as another measure of increasing solvent polarity. These results indicate that increasing solvent polarity was partially quenching the delayed emission, contributing to a decrease in the overall PLQY of *p*-TPS-DMAC-TRZ. This was also related to the decrease in the contribution of delayed fluorescence to the overall photoluminescence when the solvent polarity was increased from 1,4-dioxane to dichloromethane. 

This effect of polarity on the PLQY of p-TPS-DMAC-TRZ could also be used to understand the change observed when measurements were performed on pure films or on a mixture of the polymer with the host molecule mCP. From the measurements in solution, it is clear that the simple explanation of aggregation-induced cooling due to the proximity of the polymer chains in p-TPS-DMAC-TRZ films is not sufficient to explain the decrease in PLQY of this polymer in the solid state. If that were the case, the PLQY in all solvents, in which a dilute solution was used, should yield close values. However, this was not the case, and a clear effect of the solvent polarity was observed, showing that the polarity of the excited state environment is also relevant. In the case of using mCP, together with the high energy of the triplet that makes the energy transfer from the host to the emitting unit efficient [52], the low polarity of this molecule could be beneficial for the PLQY of p-TPS-DMAC-TRZ, considering its low dipole moment [53], resembling a polarity environment similar to that of 1,4-dioxane.

### 3.5. Electrochemical Properties 

**Figure 11:**
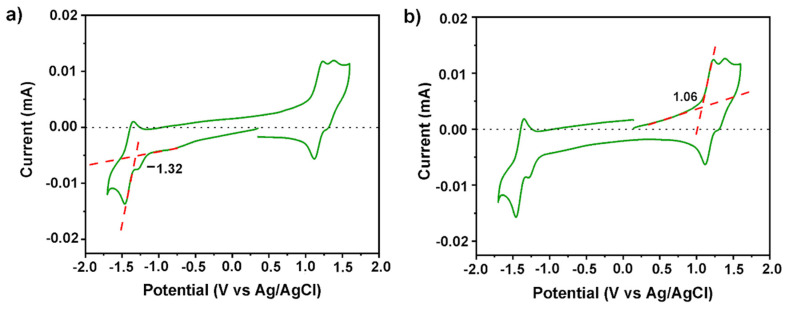
Cyclic voltammetry curves started in the negative direction (**a**) and started in the positive direction (**b**) of *p*-TPS-DMAC-TRZ. Intersection of red dashed lines indicate onset of oxidation or reduction respectively.

Cyclic voltammetry measurements for *p*-TPS-DMAC-TRZ in dichloromethane solutions were obtained starting in open circuit potential to negative direction (Figure 11a) and the same way to positive (Figure 11b). Both curves showed practically identical features, such as the reversible redox couple at negative potential values and a small cathodic pre-peak at −1.28 V, which could be related to the reduction of an adsorbed portion of the polymeric material [54]. The observed cathodic peak was attributed to the reversible reduction of the polymer. This cathodic peak (−1.44 V) was coupled with the anodic peak observed at negative potential values (−1.33 V), which evidenced the reversible nature of this redox couple at −1.39 V (E_1/2_). This couple was also observed in solutions of DMAC-TRZ, thus correlating this result with the TADF moiety of *p*-TPS-DMAC-TRZ and, most likely, to the triphenyltriazine core. 

At positive potentials, two anodic peaks at 1.25 and 1.40 V, together with a single cathodic peak at 1.13 V and a cathodic pre-peak, were observed in the CV curve (Figure 11b). The anodic peak at 1.25 V could be coupled with the anodic peak (1.13 V), forming a reversible redox couple at 1.19 V (E_1/2_), similar to that observed in DMAC-TRZ, which could be attributed to the reversible oxidation of the dimehtylacridine core of the TADF moiety. The second anodic peak, which was not observed for DMAC-TRZ, could be attributed to the TPS unit as observed in other TPS derivatives as an irreversible process [55]. 

Using the E_onset_ of oxidation in the scan started in the positive direction and the E_onset_ of reduction in the scan started in the negative direction, the HOMO and LUMO energies were estimated for the polymer (Table 2). These values were similar to those shown by DMAC-TRZ [33], which means that the TPS portion does not contribute significantly to the orbitals. This supports the idea that the TPS is a good non-conjugated unit, and as designed, does not significantly interfere with the photophysical and electrochemical properties of the TADF core. This also means that *p*-TPS-DMAC-TRZ has suitable HOMO-LUMO energy values for use as an emitting layer in an OLED device with similar materials as used by DMAC-TRZ.

The obtained electrochemical band gap (E_g_^elec^) of 2.38 eV was significantly different from the optical band gap (3.11 eV, Table 1). This fact could be attributed to the polymeric nature of the material, in which π-stacking interactions may be differently accounted for in both methods [56]. E_g_^elec^ value was lower than that reported for DMAC-TRZ (2.52 eV) [33], which could be attributed to the extension in the conjugation path described before for the dimethylacridine donor moiety. This idea gains strength considering that E_LUMO_ values of *p*-TPS-DMAC-TRZ and DMAC-TRZ were very similar (−2.76 eV and −2.78 eV, respectively), and the E_HOMO_ values differed a bit more (−5.14 eV and −5.30 eV, respectively).

### 3.6. Morphological Properties

**Figure 12:**
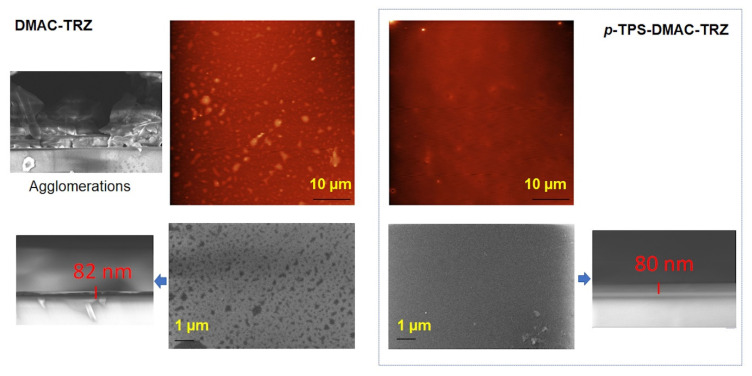
AFM and FE-SEM micrographs of DMAC-TRZ (left) and *p*-TPS-DMAC-TRZ (right) films over PEDOT:PSS coated glass substrates, with their respective 60° tilted cross-sections.

The morphological characteristics of the *p*-TPS-DMAC-TRZ films were evaluated and compared with those of DMAC-TRZ by AFM and FE-SEM (Figure 12). DMAC-TRZ films showed a roughness of 18.56 nm, while the *p*-TPS-DMAC-TRZ films evidenced a smoother surface with a roughness of 3.93 nm. These results showed that *p*-TPS-DMAC-TRZ had better morphological characteristics in the film state than DMAC-TRZ when both materials were processed with in-solution methods, such as spin-coating. This fact could be due to the polymeric nature of p-TPS-DMAC-TRZ and the presence of the tetraphenylsilane unit, which is known to promote a smooth film morphology due to its tetrahedral geometry that decreases intermolecular interactions [29]. Both films were close to ~81 nm thick (FE-SEM, tilted cross section), which was in the range of typical thicknesses of light-emitting layers in OLED devices. These images also show that the DMAC-TRZ films prepared by spin-coating had considerable agglomerations and defects compared to the smoother *p*-TPS-DMAC-TRZ films.

## 4. Conclusions

A new polymer (*p*-TPS-DMAC-TRZ) was designed and synthesized according to a non-conjugated unit TADF main chain polymer strategy, in which the TADF unit was linked with a conjugation-breaking unit through the main chain. *p*-TPS-DMAC-TRZ was prepared through a poly-Suzuki-Miyaura coupling of a 2,7-dibrominated derivative of DMAC-TRZ with a diboronic-ester monomer containing the TPS backbone unit. The polymer was soluble in organic solvents such as toluene, chloroform, dichloromethane, and THF at room temperature, which is beneficial for in-solution deposition methods to obtain thin films. The average length of the chains was 11 monomeric units with a low PDI. *p*-TPS-DMAC-TRZ showed high thermal stability with a TDT_5%_ 176 °C higher than that of DMAC-TRZ, and its T_g_ was 174 °C greater than the small molecule. This thermal behavior is highly desired in materials that make up OLED devices. As for the photophysical properties, the absorption maxima of *p*-TPS-DMAC-TRZ were blue-shifted compared to DMAC-TRZ from 390 nm to 348 nm, while the photoluminescence maxima were red-shifted from 500 nm to 508 nm in the solid state. This indicates that photophysical properties were slightly modified from the small molecule to the polymeric material. In addition, UV-Vis and PL measurements in five solvents of different polarities showed a bathochromic shift of the emission maxima when the solvent polarity was increased, demonstrating the ICT character of the excited state responsible for the light emission, which is an intrinsic feature of TADF materials. TRPL spectra at 298 K and 77 K demonstrated that the polymer retained TADF behavior where a noticeable decrease in the delayed component of the emission was observed at low temperature. PLQY of the polymer decreased considerably compared to TADF molecule from 0.83 to 0.29 in neat film, maintaining a value that resembles PLQY of similar TADF polymers. However, when used in combination with the host molecule mCP in a 1% blend film, the PLQY increased considerably, reaching a value of 0.62, which increases the potential use of *p*-TPS-DMAC-TRZ as a light-emitting doped layer in OLED devices. Furthermore, measurements of TRPL in solutions purged with N_2_ or in-air showed a significant decrease in both lifetime and delayed contribution to total photoluminescence when oxygen was present, demonstrating the involvement of the triplet state in the overall light emission mechanism, which in TADF materials participates by the reconversion of these non-emissive triplet states to the singlet state and hence delayed emission. A polarity effect was observed in which increasing the polarity of the solvent decreased the PLQY of *p*-TPS-DMAC-TRZ in solution, especially affecting the delayed component of the emission, as could be seen by the notorious decrease in the lifetime and the contribution of the delayed emission. This effect could better explain the observed PLQY of *p*-TPS-DMAC-TRZ in solid state than the simple aggregation-induced quenching due to the proximity of the excited states, where the low polarity of the mCP would be beneficial to increase this parameter, whereas the higher polarity environment encountered by the excited states of *p*-TPS-DMAC-TRZ in pure films would be detrimental. Electrochemical studies by cyclic voltammetry showed some similar patterns, such as the two reversible redox couples observed in DMAC-TRZ. However, a few additional features were observed, such as a pre-peak reduction attributed to partial adsorption of the polymer in the working electrode and an additional oxidation peak related to the TPS oxidation. Significantly, few changes were observed in the HOMO-LUMO energy values compared to DMAC-TRZ, with only a slight increase in the energy of the HOMO orbital, while the LUMO energy was practically the same. Finally, the surface morphology of DMAC-TRZ and *p*-TPS-DMAC-TRZ coated films (~80 nm) observed by AFM and FE-SEM showed that *p*-TPS-DMAC-TRZ films have smoother surfaces with fewer aggregations, which is beneficial for their potential use as a light-emitting layer. In summary, *p*-TPS-DMAC-TRZ showed better thermal and morphological properties than DMAC-TRZ when processed by in-solution methods such as spin-coating, both attributable to the polymeric nature of *p*-TPS-DMAC-TRZ and the presence of the tetraphenylsilane unit. Some photophysical properties were partially preserved, such as emission maxima and TADF behavior. However, the PLQY in the pure films decreased markedly, indicating that the design strategy should be improved in this specific aspect to maintain the improved thermal and morphological properties and, at the same time, improve or preserve the photophysical properties of the TADF core. However, when used in a host-guest-host mixture, p-TPS-DMAC-TRZ showed a markedly improved PLQY that, together with the aforementioned improved physical properties, makes it a good candidate for use as a doped light-emitting layer for solution-processed OLED devices. 

## Figures and Tables

**Table 1:** Photophysical properties of *p*-TPS-DMAC-TRZ.

In Solution λ_abs_^max^_/_ λ_em_^max^ (nm)				
Toluene	1,4-Dioxane	THF	Chloroform	Dichloromethane
349/513	348/542	348/578	349/581	350/604
In film (contribution)				
λ_em,max_ (nm)	Ε_g_^opt^ (eV) ^a^	τ_77 K_ (ns)	τ_298 K_ (ns)	PLQY
508	3.11	24.6 (0.80),	23.6 ns (0.36),	0.29
		180 (0.20)	0.290 μs (0.14),	0.62 ^b^
			2.06 μs (0.50)	
PL Lifetimes in N_2_ Solution of *p*-TPS-DMAC-TRZ (Contribution) Prompt (ns) Delayed (μs)				
Toluene	1,4-Dioxane	THF	Chloroform	Dichloromethane
19.7 (0.55) 3.81 (0.45)	27.8 (0.41) 2.49 (0.59)	23.5 (0.56) 0.833 (0.44)	21.4 (0.64) 0.724 (0.36)	15.26 (0.74) 0.437 (0.26)
PL Lifetimes (ns) in Air Solution of *p*-TPS-DMAC-TRZ (Contribution)				
Toluene	1,4-Dioxane	THF	Chloroform	Dichloromethane
11.1 (0.86) 20.3 (0.14)	19.9 (0.92) 99.0 (0.08)	13.4 (0.86) 58.8 (0.14)	13.4 (0.82) 100.0 (0.18)	12.1 (0.85) 109.0 (0.15)
PLQY ^c^ in Solution of *p*-TPS-DMAC-TRZ N_2_/Air				
Toluene	1,4-Dioxane	THF	Chloroform	Dichloromethane
0.45/0.25	0.66/0.42	0.29/0.15	0.20/0.14	0.16/0.13
Prompt and Delayed PLQY of *p*-TPS-DMAC-TRZ in N_2_ Purged Solution				
Toluene	1,4-Dioxane	THF	Chloroform	Dichloromethane
0.25/0.20	0.27/0.39	0.16/0.13	0.13/0.07	0.12/0.04

^a^ Optical band gap obtained using 1242/λ_onset_ (λ_onset_ from the solution UV-Vis spectra in toluene). ^b^ Solid state blend of 1% *p*-TPS-DMAC-TRZ in mCP. ^c^ PLQY determined using quinine bisulfate (0.5 M in sulfuric acid) as standard (PLQY = 0.546).

**Table 2:** Electrochemical properties of *p*-TPS-DMAC-TRZ.

E_red_ (V)	E_ox_ (V)	E_HOMO_^a^ (eV)	E_LUMO_^b^ (eV)	E_g_^elec c^ (eV)
−1.32	1.06	−5.14	−2.76	2.38

^a^ Calculated from (−e[E_onset_^ox^ + (4.80 − E_Fc/Fc_^+^)]). ^b^ Calculated from (−e[E_onset_^red^ + (4.80 − E_Fc/Fc_^+^)]) [56]. ^c^ Electrochemical band gap obtained from E_LUMO_-E_HOMO_.

## Data Availability

The data that support the findings of this study are available from the corresponding author upon reasonable request.
